# Supplementary material for: A versatile web app for identifying the drivers of COVID-19 epidemics
Source: J Transl Med. 2021 Mar 16;19:109. doi: 10.1186/s12967-021-02736-2 (PMC7962635; doi:10.1186/s12967-021-02736-2)
Supplement: Supplementary file 1 — Additional file 1: A Additional file is provided containing: Appendix A, details of a continuous-time version of our SCLAIV model; Appendix B, our discrete-time implementation of this model; Appendix C, parameter estimation and simulation procedures, including details of our MCMC computation; and Appendix D, additional case-study figures. [file 12967_2021_2736_MOESM1_ESM.pdf]

APPENDICES

Getz, Salter, Luisa Vissat, & Horvitz
A Versatile Web App for Identifying the Drivers of COVID-19 Epidemics

A Model Structure

The model we present here is an explicit contact compartmental version of the standard SEIR model that also includes, as found in several other COVID-19 models, both asymptomatic (A) and symptomatic (I) infections stages [17], as well as divides the exposed (E) stage into contact (C) and latent (L) disease stages. The contact compartment represents individuals that have been exposed to the pathogen, some of who return to the susceptible stage S having thwarted the infection through physical, physiological, and innate immunological mechanisms. Others in C succumb to infection and go on to the latent disease stage L (i.e., stage E in SEIR models). This division of E into C and L stages makes the contact process explicit rather than implicit, thereby allowing the effects of contact tracing to be explicitly incorporated into the dynamic model. Also, as discussed elsewhere [11], we make explicit that individuals sometimes lumped together in class R in SEIR models, have either recovered with immunity (i.e., this is the natural counterpart to a vaccinated class) in class V or are dead in class D. Finally, we include a set of SCLAI shadow or response compartments to hold individuals impacted by the processes, or drivers, used to manage the outbreak, including social distancing and subsequent social relaxation, quarantining in response to contact tracing, isolating/treating infectious individuals, and rolling out vaccination programs once suitable vaccines become available (Fig. A.1). In addition, our model makes explicit the role of surveillance and reduced transmission associated with individuals in the response compartments of our SCLAIV+response model.

A.1 Contact Explicit Dynamic Model Formulation

In presenting the details of our SCLAIV+response model in this section, we emphasize that as with all basic SEIR models, populations are assumed to be well-mixed (i.e. any individual is equally likely to come in contact with any other individual in the population—so no relevant spatial structure) and are homogeneous from a behavioral and epidemiological characteristics point of view. In addition, we assume that natural birth and death processes—other than a disease induced process—cause population changes at time scales much slower than the epidemiological process under consideration and, hence, these basic demographic processes can be ignored.

Throughout this paper, we use the roman fonts S, C, L, A, I, V and D to name the disease classes defined below, while italic fonts S, C, L, A, I, V and D refer to the actual variables representing the number of individuals in these corresponding classes. We also introduce response classes $\mathrm{S_r}$, $\mathrm{C_r}$, $\mathrm{L_r}$, $\mathrm{A_r}$, $\mathrm{I_r}$, and $\mathrm{V_r}$, with variables S_r, C_r, L_r, A_r, I_r, and V_r to respectively represent the number of individuals in each of these classes.

A.1.1 Disease classes

Our SCLAIV+response+D model consists of the following 13 variables (SCLAIV is 6, response is 6, D is 1) with flow connections among classes depicted in Fig. A.1.

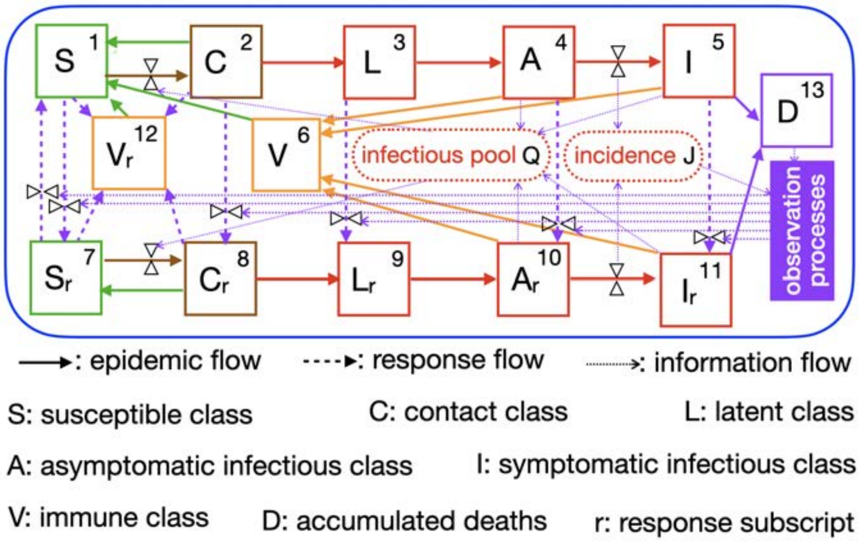

Figure A.1: An epidemic and response flow diagram of a nuanced SEIR process that we refer to as a SCLAIV+response process. Within the infectious pool Q are two parameters that account for reduced transmission of asymptomatic compared with symptomatic individuals, as well as reduced transmission of individuals in response classes A_r and I_r compared with the original A and I classes. Within the incidence pool J, is a surveillance parameter that allows us to account for the fact that not all cases are observed. See text for more details on these parameters.

1. S: number of susceptible individuals with pre-epidemic behavior
2. C: number of susceptible individuals who recently had contact with an infected individual
3. L: infected individuals in the latent/incubation stage (E class in SEIR models)
4. A: infectious individuals who are asymptomatic (they can next become either symptomatic or immune)
5. I: infectious individual who are symptomatic
6. V: recovered individuals with natural immunity (R class in SEIR model when mortality is not included)
7. S_{r}: number of susceptible individuals with behavioral modifications in response to ongoing epidemic
8. C_{r}: number of individuals in the C class who are quarantined
9. L_{r}: exposed individuals who are quarantined
10. A_{r}: infectious asymptomatic individuals who are quarantined
11. I_{r}: infectious symptomatic individuals who are treated/hospitalized/isolated
12. V_{r}: vaccinated individuals
13. D: individuals who have died from the disease (this is an absorbing state of the model and so, for clarity, is listed last)

A.1.2 Embedded SCLAIV+response+D formulation

Our 13-variable SCLAIV+response+D model, apart from two flows, can be expressed in terms of a general "donor controlled" system where all per-capita flows out of compartments are either constant or time varying. The exceptions are the two contact processes in which individuals are

transferred from S to C and from S_r to C_r. These two flows, as we will see below, depend both on the value of the recipient compartment and the size of the active population as a whole (all individuals minus those that have died).

Continuous-time, donor-controlled systems of equations can be compactly expressed in terms of a vector of system's variables $\mathbf{x} = (x_1, ..., x_n)'$ ($'$ denotes vector transpose), and a matrix F of elements f_{ij}, $i, j = 1, ..., n$ by the equation

$$\frac{d\mathbf{x}}{dt} = \mathbf{F}\mathbf{x} \tag{A.1}$$

where the elements f_{ij}, $i, j = 1, ..., n$, expressed in terms of the donor controlled per-capita flow rates γ_{ij}, (i.e. flows out of compartment i into compartment j),

$$\begin{aligned} f_{ii} &= -\left(\sum_{j=1}^{n} \gamma_{ji}\right), \qquad i = 1, ..., n, \\ f_{ij} &= \gamma_{ij}, \quad j \neq i,\ i, j = 1, ..., n. \end{aligned} \tag{A.2}$$

Under the assumption that no intrinsic growth or decay process occur within any of the disease classes (i.e., no births, non-disease induced mortality, or migration processes are included), it follows that $\gamma_{ii} = 0$, $i = 1, ...n$. In this case, the equation for the ith variable in systems Eq. A.2 is

$$\frac{dx_i}{dt} = \sum_{j \neq i,\, j=1}^{n} \gamma_{ij} x_j - \left(\sum_{j=1}^{n} \gamma_{ji}\right) x_i, \quad i = 1, ..., n \tag{A.3}$$

If all the rates in the system flow matrix Γ, with ij^{th} element

$$(\Gamma)_{ij} = \gamma_{ij},\ i, j = 1, ..., n, \quad \text{where} \quad \gamma_{ii} = -\sum_{j=1}^{n} \gamma_{ji},\ i = 1, ...n$$

are constant or time dependent then the system is linear. Nonlinearities enter when one or more of the rates are dependent on the values of the various systems variables. Whether completely linear or not, we note that this system is subject to conservation principle invoked by the relationship $\sum_{i=1}^{13} x_i(t) = \sum_{i=1}^{13} x_i(0)$ for all t because under the "no intrinsic growth or decay process" assumption it follows that

$$\sum_{i=1}^{13} \frac{dx_i}{dt} = 0 \Longrightarrow \sum_{i=1}^{13} x_i(t) = \sum_{i=1}^{13} x_i(0) \quad \text{for all } t \geq 0 \tag{A.4}$$

Letting γ_{ij} represent the flow from compartment j to compartment i and using the numbering system of the variables listed above for our SCLAIV+response+D mode, we have $13 \times 13 = 169$ potential flows, but only 28 of the off-diagonal entries are non-zero, as indicated in the flow matrix Γ below . Note that the last column of this matrix contains all zeros because D (x_{13}, in the notation of Eq. A.3), is an absorbing compartment (no outflows, only inflows) and the remaining 12 diagonal elements are the negative sum of the rest of the columns in which they appear because the system satisfies Eq. A.4.

$$
\Gamma = \begin{pmatrix}
-\sum\limits_{i=2,7,12}\gamma_{i1} & \gamma_{1\,2} & 0 & 0 & 0 & \gamma_{1\,6} & \gamma_{1\,7} & 0 & 0 & 0 & 0 & \gamma_{1\,12} & 0 \\
\gamma_{2\,1} & -\sum\limits_{i=1,3,8,12}\gamma_{i2} & 0 & 0 & 0 & 0 & 0 & 0 & 0 & 0 & 0 & 0 & 0 \\
0 & \gamma_{3\,2} & -\sum\limits_{i=4,9}\gamma_{i3} & 0 & 0 & 0 & 0 & 0 & 0 & 0 & 0 & 0 & 0 \\
0 & 0 & \gamma_{4\,3} & -\sum\limits_{i=5,6,10}\gamma_{i4} & 0 & 0 & 0 & 0 & 0 & 0 & 0 & 0 & 0 \\
0 & 0 & 0 & \gamma_{5\,4} & -\sum\limits_{i=6,11,13}\gamma_{i5} & 0 & 0 & 0 & 0 & 0 & 0 & 0 & 0 \\
0 & 0 & 0 & \gamma_{6\,4} & \gamma_{6\,5} & -\gamma_{1\,6} & 0 & 0 & 0 & \gamma_{6\,10} & \gamma_{6\,11} & 0 & 0 \\
\gamma_{7\,1} & 0 & 0 & 0 & 0 & 0 & -\sum\limits_{i=1,8,12}\gamma_{i7} & \gamma_{7\,8} & 0 & 0 & 0 & 0 & 0 \\
0 & \gamma_{8\,2} & 0 & 0 & 0 & 0 & \gamma_{8\,7} & -\sum\limits_{i=7,9,12}\gamma_{i8} & 0 & 0 & 0 & 0 & 0 \\
0 & 0 & \gamma_{9\,3} & 0 & 0 & 0 & 0 & \gamma_{9\,8} & -\gamma_{i10\,9} & 0 & 0 & 0 & 0 \\
0 & 0 & 0 & \gamma_{10\,4} & 0 & 0 & 0 & 0 & \gamma_{10\,9} & -\sum\limits_{i=6,11}\gamma_{i10} & 0 & 0 & 0 \\
0 & 0 & 0 & 0 & \gamma_{11\,5} & 0 & 0 & 0 & 0 & \gamma_{11\,10} & -\sum\limits_{i=6,13}\gamma_{i11} & 0 & 0 \\
\gamma_{12\,1} & \gamma_{12\,2} & 0 & 0 & 0 & 0 & \gamma_{12\,7} & \gamma_{12\,8} & 0 & 0 & 0 & -\gamma_{1\,12} & 0 \\
0 & 0 & 0 & 0 & \gamma_{13\,5} & 0 & 0 & 0 & 0 & 0 & \gamma_{13\,11} & 0 & 0
\end{pmatrix} \tag{A.5}
$$

Our basic SCLAIV process is limited to the flows among the first 6 compartments (the top left 6×6 submatrix of Γ defined in Eq. A.5), which are assumed constant except for $\gamma_{2\,1}$, the contact process flow from S to C (i.e., x_1 to x_2 in the notation of Eq. A.3). This flow is assumed to depend on either direct contact with infectious individuals in disease class A and I (i.e., x_4 and x_5 in the notation of Eq. A.3)—or, more generally, with an infectious pool Q to which infectious individuals contribute, because some contacts are with viral-laden fomites rather than infectious individuals per se [18] (i.e. accounting for both direct and indirect modes of transmission, as discussed elsewhere [1, 3]). In a so-called frequency-dependent, rather than mass-action-dependent contact process, the intensity of the pool depends on the proportion of infectious individuals in the population, rather than the total number of infectious individuals [10]. Similarly, in the broader the SCLAIV+response+D system, the flow $\gamma_{8\,7}$ from S_r (x_7) to C_r (x_8) is assumed to have this same frequency dependence, but is scaled down by a factor that accounts for reduced contact and, hence, transmission rates in social distancing individuals. The specifics of our formulation of the infectious pool Q, in terms of the number of individuals in classes A, I, A_r, I_r and a total active (living) population variable N are provided by Eq. A.16 below.

A.1.3 Contact rates and population size

In SEIR models, the processes of pathogen transmission and infection (i.e., successful pathogen invasion of a host once exposed to the pathogen) are concatenated and represented by a single expression $\beta\phi\big(S(t), I(t), N(t)\big)$, where β is a force of transmission parameter and ϕ some appropriate functional form [15]. Specifically $\phi = \frac{SI}{N}$ when assumed to be frequency dependent [10]. In our SCLAIV model, the parameter β is now separated out into the product of i) a force of contact rate parameter κ and ii) a proportion of individuals that succumb to (as opposed to thwarting) the

infection once exposed to the pathogen (expression developed in Section B).

In SEIR models, $N(t) = S(t) + I(t) + E(t) + R(t)$ provided $R(t)$ does not include individuals that have died from the disease. In our SCLAIV+response model, the comparable expression is

$$N(t) = S(t) + C(t) + L(t) + A(t) + I(t) + V(t) + S_{\rm r}(t) + C_{\rm r}(t) + L_{\rm r}(t) + A_{\rm r}(t) + I_{\rm r}(t) + V_{\rm r}(t) \quad (A.6)$$

Before the infection begins, however, the size of the population at risk is often not known. Further, short of considering a closed population on an island, almost all populations "leak": movement in and out occurs to some extent. Thus, when considering the size of the population at risk to COVID-19 in, say, London, New York, or Rio de Janeiro, a precise value to the size of the population at risk cannot be assigned. The solution to this problem is to normalize the analysis by standardizing the population size to a nominal value, say, $N_{\rm nom} = 10^5$ [2, 5] or $N_{\rm nom} = 10^7$ (as we do here for larger outbreaks), and then citing results per hundred thousand individuals (or per 10 million).

As long as the relative number of infected individuals in the population remains low, the number of new cases remains largely independent of the size of the population at risk. Thus, epidemics start out generating new cases at similar rates in small towns and large cities, as long as the characteristic contact rates and epidemiological parameters in these different settings are similar. It is only once the epidemic gets going that towns run out of susceptibles much more rapidly than large cities and the epidemics in these contrasting situations begin to look quite different. In small towns the epidemic gets extinguished much earlier because it runs out of susceptibles, while in large cities it can continue to grown exponentially for a longer period of time. For this reason it is better to set $N_{\rm nom} = 10^7$ when modeling COVID-19 outbreaks in large cities or medium to large countries, provided this value is smaller than the true size of the population at risk, and the proportion of individuals in class V (a measure of the relative size of the epidemic) is less than, say, 10%.

A.2 Flows Rates

A.2.1 Basic SCLAIV process

An SEIR epidemiological process model, where R actually consists of both V (immune) and D (dead), is characterized by four parameters: the flows from S to E, E to I, I to V and a disease induced mortality flow from I to D. A fifth parameter is needed when the SEIR process is extended to the more general SEIRS formulation [14] (i.e., immunity is lost over time as individuals flow back from V to S). In a SCLAIV process, the splitting of E (exposed) into C (contact) and L (latent), and the addition of an A (asymptomatic) class now raises the number of parameters to the following eight:

1. the *contact rate* parameter $\kappa > 0$ scales the per-capita flow rate of susceptibles from class S to C in proportion to the per-capita intensity of the infectivity pool, where the reduced contribution to the pool by asymptomatic individuals, A, compared with symptomatic individuals, I, is scaled by an *infectivity reduction* parameter $\varepsilon \in [0, 1)$. Specifically, the per-capita flow from S to C in the case of the SCLAIV model alone, for which the extant population is $N(t) = S(t) + C(t) + L(t) + A(t) + I(t) + V(t)$, is

$$\underline{\text{SCLAIV alone:}} \qquad \gamma_{21} = \frac{\kappa\big(I(t) + \varepsilon A(t)\big)}{N(t)} \quad (A.7)$$

(Note: in an SEIR model $E(t) = C(t) + L(t)$, $A(t) = 0$ and $V(t) \equiv R(t)$)

2. the *succumb period* parameter $\pi_{\rm suc} \geq 0$ (i.e., pertaining to individuals who, after contact with the pathogen, succumb to infection), whose inverse is the per-capita flow rate

$$\gamma_{3\,2} = 1/\pi_{\rm suc} \tag{A.8}$$

scales the flow from C to L

3. the *thwart period* parameter $\pi_{\rm thw} \geq 0$ whose inverse is the per-capita flow rate

$$\gamma_{1\,2} = 1/\pi_{\rm twa} \tag{A.9}$$

scales the flow from C back to S (i.e., those individuals who, after contact with the pathogen, thwart its invasion)

4. the *latent period* parameter $\pi_{\rm lat} \geq 0$, whose inverse is the per-capita flow rate

$$\gamma_{4\,3} = 1/\pi_{\rm lat} \tag{A.10}$$

scales the flow from L to A

5. the *asymptomatic period* parameter $\pi_{\rm asy} > 0$, whose inverse is the per-capita flow rate

$$\gamma_{5\,4} = 1/\pi_{\rm asy} \tag{A.11}$$

scales the flow from A to I.

6. the *infectious/recovery period* parameter $\pi_{\rm rec} > 0$, whose inverse is the per-capita flow rate

$$\gamma_{6\,5} = 1/\pi_{\rm rec} \tag{A.12}$$

scales the flow from I to V. To keep things simple, we assume that the per-capita flow from A to V is scaled also by this same parameter i.e.,

$$\gamma_{6\,4} = 1/\pi_{\rm rec} \tag{A.13}$$

which implies that asymptomatic individuals can either play the role of presymptomatic individuals moving on to become symptomatic at a rate scaled by $1/\pi_{\rm asy}$ or can recover from being asymptomatic at a rate scaled by $1/\pi_{\rm rec}$

7. the *immune period* parameter $\pi_{\rm imm} \geq 0$, whose inverse is the per-capita flow rate

$$\gamma_{1\,6} = 1/\pi_{\rm imm} \tag{A.14}$$

scales the flow from V back to S as immunity wanes over time

8. the *disease induced mortality rate* parameter $\alpha \geq 0$ is the per-capita flow rate from I to the proportion of D that represents in our SCLAIV+D process those dying from the disease: that is

$$\gamma_{13\,5} = \alpha \tag{A.15}$$

A.2.2 SCLAIV response process

The SCLAIV+response process can be divided along the following lines: 1.) a flow chain of individuals from $\mathrm{S_r}$ to $\mathrm{I_r}$ through $\mathrm{C_r}$, $\mathrm{L_r}$ and $\mathrm{A_r}$ and then onto V and D that parallels the SCLAIV+D process (Fig. A.1); 2.) flows from basic disease classes to corresponding response classes (e.g., S to $\mathrm{S_r}$, C to $\mathrm{C_r}$ etc.—see Fig. A.1) through the implementation of drivers described in the next subsection, used to develop policies for control and elimination of the outbreak. All but one of the first set of flows are governed by the same per-capita flow rates as in the basic SCLAIV process, the exception being the flow of individuals from $\mathrm{S_r}$ to $\mathrm{C_r}$ due to the reduced contact that individuals in $\mathrm{S_r}$ have with the infectious pool Q, compared with the contact rates of S with Q.

The notion of a "virtual infectious pool" variable Q (tantamount to a measure of the amount of pathogen available for infecting susceptibles) allows us to represent the risk of making contact with the pathogen both directly (individual to individual) and indirectly (individual contacting a fomite) under the following two assumptions. First, four classes of individuals—A, $\mathrm{A_r}$, I, and $\mathrm{I_r}$—contribute to this infectious pool. Second, asymptomatic individuals (A, $\mathrm{A_r}$) shed only a proportion $\varepsilon \in [0, 1)$ of pathogens that symptomatic individuals (I, $\mathrm{I_r}$) shed. Under these assumptions, a measure of the size/intensity of the infectious pool is

$$Q(t) = \varepsilon\Big(A(t) + A_r(t)\Big) + I(t) + I_r(t) \tag{A.16}$$

The per-capita rates at which susceptible individuals encounter this pool at time t is now assumed to be proportional to the of size of the pool $Q(t)$ normalized by number of individuals $N(t)$ (Eq. A.6) available for contact. We also let $\delta_{\mathrm{con}} \in [0, 1)$ represent the extent to which social distancing behavior reduces contact with infectious pool Q. In this case, equation A.7 can be generalized to obtain the flow rates

$$\underline{\text{SCLAIV+response:}} \qquad \gamma_{2\,1}(t) = \frac{\kappa Q(t)}{N(t)} \quad \text{and} \quad \gamma_{8\,7}(t) = \frac{\delta_{\mathrm{con}}\kappa Q(t)}{N(t)} \tag{A.17}$$

For the sake of completeness, the assumption that remaining response compartments are the same as in the basic SCLAIV process implies that

$$\gamma_{9\,8} = 1/\pi_{\mathrm{suc}}, \gamma_{7\,8} = 1/\pi_{\mathrm{twa}}, \gamma_{10\,9} = 1/\pi_{\mathrm{lat}}, \gamma_{11\,10} = 1/\pi_{\mathrm{asy}}, \gamma_{6\,10} = 1/\pi_{\mathrm{rec}}, \ \& \ \gamma_{6\,11} = 1/\pi_{\mathrm{rec}} \tag{A.18}$$

and, for simplicity, we also assume that

$$\gamma_{13\,11} = \alpha \tag{A.19}$$

In reality, the mortality rates in compartments I and $\mathrm{I_r}$ are bound to differ, but the change in mortality rate over time is, perhaps, better dealt with as a driver that leads to a reduction in mortality rates once the medical establishment has improved its protocols and therapeutics for treating individuals infected with the disease. Note, under the assumption that the surveillance and treatment/isolation drivers are activate from the start of the epidemic, during the course of an outbreak most individuals land up in compartment $\mathrm{I_r}$ rather than I itself. On the other hand, individuals will not be shunted to compartments $\mathrm{C_r}$, $\mathrm{L_r}$ and $\mathrm{A_r}$ unless contact tracing along with quarantining is implemented.

A.3 Response Drivers

The response drivers, which are responsible for transfer individuals from the SCLAIV set of classes to the SCLAIV-response classes (Fig. A.1) can either be specified to have some constant value or may be represented in terms of switching curves when time varying. Specifically, when represented in terms of switching curves, we assume that the drivers are 0 until they are initiated at some point in time, either at the start of the outbreak or sometime into the outbreak, and that they may either represent a gearing up process, as in surveillance, social distancing and quarantining, or a gearing down process, as the relaxation of social distancing and reductions in disease-induced mortality rates.

The classic curve for modeling a graduated switch from a value of 0 to 1 on the infinite line $(-\infty, \infty)$ is the sigmoidal or logistic curve with two parameters: the first specifies the switch point (i.e., the point of inflection on the curve) and the second specifies the slope of the curve at the switching point. This function involves the exponential that, when replaced with a power function, switches on $[0, \infty)$. This new related function can be rescaled using two additional parameters and translated by a constant for the addition of one more parameter to have it turn on at some time t_0 and switch between initial and final values at some switching time $t_{1/2}$ (Fig. A.2). Specifically, in the context of a particular driver named "x" we define a function that switches between two values $\delta_{\mathrm{x}\,0}$ and $\delta_{\mathrm{x}\,\infty}$ on the interval the half-infinite interval $[t_{\mathrm{x}\,0} \geq 0, \infty]$ by the equation

$$\delta_{\mathrm{x}}(t) = \begin{cases} 0 & \text{for} \quad t \in [0, t_{\mathrm{x}\,0}) \\ \dfrac{\delta_{\mathrm{x}\,0} + \delta_{\mathrm{x}\,\infty}\,\mathcal{T}_{\mathrm{x}}\,(t - t_{\mathrm{x}\,0})}{1 + \mathcal{T}_{\mathrm{x}}(t - t_{\mathrm{x}\,0})} & \text{for} \quad t \geq t_{\mathrm{x}\,0}, \quad \text{where } \mathcal{T}_{\mathrm{x}}(t) = \left(\dfrac{t}{t_{\mathrm{x}\,1/2}}\right)^{\sigma_{\mathrm{x}}} \end{cases} \tag{A.20}$$

We note that for this driver, the time scaling function $\mathcal{T}_{\mathrm{x}}(t)$ implies that the switch is half-way complete at time $t = t_{\mathrm{x}\,1/2}$, provided $t_{1/2} \geq t_0$, and that the abruptness of this switch (i.e. steepness at $t_{\mathrm{x}\,1/2}$) is controlled by the value σ_{x} [6]: as $\sigma_{\mathrm{x}} \to \infty$ the switch approaches a step function that switches from $\delta_{\mathrm{x}\,0}$ to $\delta_{\mathrm{x}\,\infty}$ at $t = t_{\mathrm{x}\,1/2}$. The function is still defined and valid for the case $t_{\mathrm{x}\,1/2} < t_{\mathrm{x}\,0}$, but note we have specified the function to be zero for $t < t_{\mathrm{x}\,0}$. Typically $\sigma_{\mathrm{x}} \in [2, 20]$ is sufficient to go from a gradual switch to an almost instantaneous switch (Fig. A.2). Also, if $\delta_{\mathrm{x}\,0} < \delta_{\mathrm{x}\,\infty}$ then curve switches up (on) otherwise it switches down (off). Also, if we want to ensure that $\frac{d\delta_{\mathrm{x}}}{dt} = 0|_{t=t_{\mathrm{x}\,0}}$, then we need to select $\sigma_{\mathrm{x}} > 1$.

In the formulation of our model, we allow the following parameters/flow-rates to either be constant (usually when fitting initial conditions and contact and transmission rate parameters in the model to the initial outbreak phase) or to be represented as drivers that can be switched up or down as a means to managing an outbreak. When represented as switching functions, each of the drivers below follows Eq. A.20 with x replaced by a three letter acronym as indicated.

Surveillance response driver, $\delta_{\mathrm{sur}}(t)$, determines the proportion of new cases identified in each time period through an equation presented below in the context of our discrete time analogue of the continuous time model that has the flow topology of equation A.5.

Social distancing driver, $\delta_{\mathrm{sod}}(t) \in [0, 1)$, determines the per-capita flow rate from the susceptible class S to $\mathrm{S_r}$: i.e., $\gamma_{7\,1} = \delta_{\mathrm{sod}}(t)$

Social relaxation driver, $\delta_{\mathrm{sor}}(t)$, is used to set the flow rate $\gamma_{1\,7}$ from susceptible class $\mathrm{S_r}$ back to S using the relationship $\gamma_{1\,7} = \delta_{\mathrm{sor}}(t)$

Quarantine response driver, $\delta_{\mathrm{qua}}(t)$, determines the per-capita flow rates $\gamma_{8\,2}$, $\gamma_{9\,3}$, and $\gamma_{10\,4}$ from classes C, L and A to $\mathrm{C_r}$, $\mathrm{L_r}$ and $\mathrm{A_r}$ respectively: i.e., $\gamma_{8\,2} = \gamma_{9\,3} = \gamma_{10\,4} = \delta_{\mathrm{qua}}(t)$

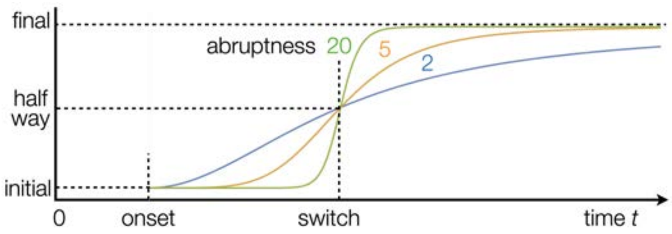

Figure A.2: Switching curves (Eq. A.20) used to represent the time-course of drivers included in our SCLAIV response process are 0 until onset time t_0 at an initial value (δ_0; dropping the "x" designator in Eq. A.20). They then switch to a final value (δ_∞) by passing through the "half-way" point at time $t_{1/2}$, provided $t_{1/2} \geq t_0$, with an "abruptness" or steepness specified by the parameter $\sigma > 1$ (strict inequality ensures that the derivative of $\delta(t)$ is zero from the left at t_0) and the switch becomes increasingly sharp to become a step-function as $\sigma \to \infty$. If the initial value is larger than final value the switch is "off" rather than "on." Note, if $t_{1/2} < t_0$ then the switching curve is still valid, since it is only defined as non-zero for $t \geq t_0$, but $t_{1/2}$ can no longer be interpreted as the half-way value point.

Patient isolation/treatment driver, $\delta_{\rm iso}(t)$ determines the per-capita flow rate of symptomatic individuals in class I to patient care/treatment class $\rm I_r$: i.e., $\gamma_{11\,5} = \delta_{\rm iso}(t)$

Vaccination rate driver, $\delta_{\rm vac}(t)$, once a vaccine becomes available, will determine the per-capita flow rate at which individuals are vaccinated during the course of the epidemic: i.e., $\gamma_{8\,2} = \delta_{\rm vac}(t)$

Contact rate reduction driver, $\delta_{\rm con}(t) \in [0, 1]$, is needed to compute $\gamma_{2\,1}$ using Eq. A.17

Lastly, one can also express the disease-induced mortality rate α as a switching function, but we have not done so because we have not treated reductions to disease-induced mortality as a driver in this formulation. Rather, we view this reduction as driven by a much longer term therapeutics development process that, as yet, cannot be implemented on the time scale of the various drivers considered above.

B Discrete Time Implementation

B.1 Competing Rates Formulation

Continuous-time formulations of SEIR processes have notational advantages over discrete-time formulations in terms of presentation and the development of theory, such as the derivation of stability results, including in metapopulation situations [12], and the identification of backward bifurcation phenomena [20]. Discrete-time formulations, however, are more directly related to incidence and mortality data (reported daily in the case of COVID-19), simpler to numerically simulate, and more easily extended to stochastic settings [9,11]. Of course, individual-based models provide the greatest flexibility of all when it comes to dealing with stochasticity—particularly in the context of following transmission chains [8] or dealing with all manner of heterogeneities [21] and proliferation of diversity in the pathogen itself [19].

When epidemics are relatively large—on the order of tens of thousands or more individuals—and the focus is on developing policy for managing an outbreak rather than exploring questions relating to host and pathogen heterogeneity, then a discrete time systems formulation provides the most efficient way to undertake scenario analyses. The most consistent way to discretize a continuous systems model represented by the flow matrix Eq. A.5 is to use a competing rates approach to obtain the analogous equations [11]. Specifically, the equations so obtained are

$$x_i(t+1) = \sum_{j=1}^{13} p_{ij}(t) x_j(t) \tag{B.1}$$

$$p_{ji}(t) = \frac{\gamma_{ji}(t)\left(1 - e^{-\sum_{k=1,\,k\neq j}^{13}\gamma_{ki}(t)}\right)}{\sum_{k=1,\,k\neq i}^{13}\gamma_{ki}(t)}, \quad j=1,...,13 \quad \text{and} \quad p_{ii}(t) = 1 - \sum_{j\neq i,\,j=1}^{13} p_{ji}(t), \quad i=1,...,13 \tag{B.2}$$

where the matrix $P(t)$ with elements $p_{ij}(t),\ i,j=1,...n$, have the structure depicted in Fig. B.1.

As mentioned earlier, the force of transmission parameter β in the SEIR formulation is defined to be the per-capita contact rate κ multiplied by the proportion/probability $p_{3\,2}$ of individuals succumbing to infection having made contact with the pathogen. Using Eqs. A.8, A.9 and B.2 to express this proportion in terms of the periods π_{thw} and π_{suc}, β can now be expressed as

$$\beta \approx \frac{\kappa(1/\pi_{\text{thw}})\left(1 - e^{-(1/\pi_{\text{thw}} + 1/\pi_{\text{suc}} + \delta_{\text{qua}}(t))}\right)}{1/\pi_{\text{thw}} + 1/\pi_{\text{suc}} + \delta_{\text{qua}}(t)} = \frac{\kappa\left(1 - e^{-(1/\pi_{\text{thw}} + 1/\pi_{\text{suc}} + \delta_{\text{qua}}(t))}\right)}{1 + (\pi_{\text{thw}}/\pi_{\text{suc}}) + \pi_{\text{thw}}\delta_{\text{qua}}(t)} \tag{B.3}$$

Note we use $\approx$ rather than strict equality because β and κ are rates in continuous time formulations while the proportion succumbing to infection is computed using a competing rates approximation that applies over one unit of time (i.e., we are mixing a continuous time process with a discrete time event). Thus the estimate β will depend on the units of time used in the formulation (e.g., days for relatively fast spreading diseases versus weeks or months for slower spreading diseases).

In addition, we note that these proportions in Eq. B.2 are constants whenever the values γ_{ij} used to compute them are constant. If any $\gamma_{ij}(t)$ are time varying, either because they are computed from drivers or part of the transmission process (Eq. A.17), then as the values change they are approximated by constants over each time interval $[t, t+1)$, but change from the start of one time

interval to the next as the solution numerical unfolds (i.e., is iterated from $t = 0$ to final time $t = T$ using the transition matrix P depicted in Fig. B.1 [11]).

The conservative property of the matrix Γ, given by Eq. A.5 (i.e., all column sums are zero) implies that all columns of the transition matrix $P(t)$ (Fig. B.1) sum to 1. We can either interpret the elements of the matrix P as proportions (deterministic formulation) or as probabilities. The latter applies in the case of stochastic simulations (i.e., the matrix P becomes a "stochastic matrix") and each simulation is then one instatiation of a Markov chain process. Thus we can either use the matrix P to generate deterministic or stochastic simulations, as described in [11].

$$\mathbf{P} = \begin{pmatrix}
p_{1\,1} & p_{1\,2} & 0 & 0 & 0 & p_{1\,6} & p_{1\,7} & 0 & 0 & 0 & 0 & p_{1\,12} & 0 \\
p_{2\,1} & p_{2\,2} & 0 & 0 & 0 & 0 & 0 & 0 & 0 & 0 & 0 & 0 & 0 \\
0 & p_{3\,2} & p_{3\,3} & 0 & 0 & 0 & 0 & 0 & 0 & 0 & 0 & 0 & 0 \\
0 & 0 & p_{4\,3} & p_{4\,4} & 0 & 0 & 0 & 0 & 0 & 0 & 0 & 0 & 0 \\
0 & 0 & 0 & p_{5\,4} & p_{5\,5} & 0 & 0 & 0 & 0 & 0 & 0 & 0 & 0 \\
0 & 0 & 0 & p_{6\,4} & p_{6\,5} & p_{6\,6} & 0 & 0 & 0 & p_{6\,10} & p_{6\,11} & 0 & 0 \\
p_{7\,1} & 0 & 0 & 0 & 0 & 0 & p_{7\,7} & p_{7\,8} & 0 & 0 & 0 & 0 & 0 \\
0 & p_{8\,2} & 0 & 0 & 0 & 0 & p_{8\,7} & p_{8\,8} & 0 & 0 & 0 & 0 & 0 \\
0 & 0 & p_{9\,3} & 0 & 0 & 0 & 0 & p_{9\,8} & p_{9\,9} & 0 & 0 & 0 & 0 \\
0 & 0 & 0 & p_{10\,4} & 0 & 0 & 0 & 0 & p_{10\,9} & p_{10\,10} & 0 & 0 & 0 \\
0 & 0 & 0 & 0 & p_{11\,5} & 0 & 0 & 0 & 0 & p_{11\,10} & p_{11\,11} & 0 & 0 \\
p_{12\,1} & p_{12\,2} & 0 & 0 & 0 & 0 & p_{12\,7} & p_{12\,8} & 0 & 0 & 0 & p_{12\,12} & 0 \\
0 & 0 & 0 & 0 & p_{13\,5} & 0 & 0 & 0 & 0 & 0 & p_{13\,11} & 0 & 1
\end{pmatrix}$$

SCLAIV epidemic | response flows | SCLAIV-response sub-epidemic | disease mortality

Figure B.1: A depiction of the flow topology implied by the sparse structure of the stochastic matrix P, which can be used to carry out deterministic/stochastic simulations of the SCLAIV+response+D process when the elements p_{ij} in the matrix P are treated as proportions/probabilities of individuals transferring from disease class j into disease class i at time t. Color-coded blocks of elements pertain to transfers under the basic SCLAIV process (yellow), the parallel SCLAIV response process (pink), the SCLAIV response-driver processes (green) and the mortality process (red)

B.2 Model Fitting

Our basic SCLAIV formulation, under simplifying assumptions that we introduced along the way, requires values to be estimated for the following nine parameters introduced in Subsection A.2: κ (force of contact), ε (reduced infectiousness of asymptomatics), π_{suc} (succumb period), π_{thw} (thwart period), π_{lat} (latent period), π_{asy} (asymptomatic period), π_{rec} (recovery from infection period), π_{imm} (immune period), and α (disease induced mortality rate). In addition, the 6 initial conditions S_0, C_0, L_0, A_0, I_0, and V_0 need to be set, assuming $D_0 = 0$.

In fitting a basic SCLAIV process to a set of initial outbreak data, it may be reasonable to assume that all of the response drivers, apart from a background surveillance and isolation of the sickest patients, are switched off. Surveillance needs to be operating at some initial level $\delta_{\text{sur}\,0}$ (which either implies that $t_{\text{sur}\,0} = 0$—see Eq. A.20 or we can treat surveillance as a constant rather

than a switching function) if any cases are to be observed. If we assume in our model that, initially, a proportion $\delta_{\mathrm{sur}\,0} \in (0, 1]$ of new infectious cases are observed, then the number of new infectious cases $O(t)$ observed during time period t, expressed in terms of the proportion $p_{5\,4}$ of individuals transferring from A to I, is given by

$$\underline{\text{Initial observation process:}} \qquad O(t) = \delta_{\mathrm{sur}\,0}\, p_{5\,4} I(t) \tag{B.4}$$

Once the full SCLAIV+response process is underway, the observation process now includes the proportion $p_{11\,10}$ of individuals transferring from $\mathrm{A_r}$ to $\mathrm{I_r}$ as well. In this case the observation process, which now may depend on a surveillance driver $\delta_{\mathrm{sur}}(t)$ that is ramping up over time, is given under the assumption that the same surveillance level applies to class I_{r} (of course a different assumption can be made)

$$\underline{\text{Ongoing observation process:}} \qquad O(t) = \delta_{\mathrm{sur}}(t)\big(p_{5\,4} I(t) + p_{11\,10} I_r(t)\big) \tag{B.5}$$

A number of the SCLAIV parameters can be independently estimated directly from etiological studies of the progression of disease in individuals under observation, treatment or hospitalization. These include the parameters π_{lat}, π_{asy} and, π_{rec} (Table 1 in the paper). The reduced infectivity ε of asymptomatic cases, though somewhat more difficult to obtain directly from case studies, may also be estimated independently from measurements at the rates at which asymptomatic individuals shed pathogens relative to symptomatic individuals.

The mortality rate parameter α can be estimated directly from mortality data. It appears to vary greatly from country to country in the case of COVID-19, ranging from $> 10\%$ for some Western European countries through 5-6% in the US to $< 1\%$ for some African and Middle Eastern countries (Worldometer COVID-19 Country Data Table). Of course, the reliability of these numbers depends on many factors, including surveillance levels, healthcare infrastructure, and willingness of the country to be transparent about its ongoing epidemic.

With respect to the two parameters π_{thw} and π_{suc}, their ratio rather than their absolute values largely determines the proportion of individuals that return to S versus move onto L (cf. Eq. B.2). Thus, as a first cut, we set $\pi_{\mathrm{thw}} = 1$, to reduce by 1 the number of parameters that need to be estimated in the model.

B.3 Initial Conditions

It is uncertain how many infectious individuals are present in the population when the first case of a disease is identified in a particular population. Identification will be based on the presentation of symptoms, but will also depend on the the initial surveillance level $\delta_{\mathrm{sur}\,0}$, which cannot be estimated without considerable testing that only comes at a later stage in monitoring an outbreak. Thus the initial value I_0 will need to be estimated. Its value will depend on the initial value that we assign to surveillance. In the absence of historic data that provides some estimate for $\delta_{\mathrm{sur}\,0}$, we might want to compare fits obtained for nominal assignments $\delta_{\mathrm{sur}\,0} = 0.1$ and $\delta_{\mathrm{sur}\,0} = 0.5$, noting that the smaller the value we select for $\delta_{\mathrm{sur}\,0}$, the concomitantly larger the estimates of I_0 will be, when using Eq. B.4 to fit the model to observed incidence.

If we want to reduce the number of parameters that need to be estimated, we can roughly base initial estimates of A_0 and L_0 on I_0 as follows. If the average individual remains in I_0 for a period π_{rec} and, if during this average time π_{rec} that it takes an individual to move through the I class,

the number of individuals in I has grown by an amount G to the level GI_0 then we need to ensure that on average $G/\pi_{\rm rec}$ individuals move into the I class each unit of time during the period $\pi_{\rm rec}$. If each individual remains in A for an average period $\pi_{\rm asy}$, then we require

$$A_0 = \left(\frac{\pi_{\rm asy}}{\pi_{\rm rec}}\right) GI_0 \tag{B.6}$$

to ensure sufficient individuals move from A to meet the level of growth G. A similar argument of how many individuals are initially needed in disease class L to produce A_0 leads to the equation

$$L_0 = \left(\frac{\pi_{\rm lat}}{\pi_{\rm rec}}\right) GA_0 = \left(\frac{\pi_{\rm lat}\pi_{\rm asy}}{\pi_{\rm rec}^2}\right) G^2 I_0 \tag{B.7}$$

These estimates can always be checked to be reasonable, once the fit has been made and the initial outbreak simulated. This approximation, however, may be more useful for Bayesian MCMC [4,13], as discussed in Section C.3 than maximum likelihood (ML) estimation since since the former is much more computationally demanding than the latter.

The initial value C_0 is more complicated because, beyond flows from C to A, it depends on flows back and forth between compartments S and C, as well as flows from C to $\rm C_r$ when the quarantine driver is switched on. Thus, as with I_0, we decided to include C_0 as a member of the set of parameters whose values we would estimate by fitting model simulations to the initial set of incidence data.

When estimating parameters of an outbreak in an epidemiological naïve population, as is the case with an emerging disease such as COVID-19 (but generally not for measles or influence), it is reasonable to set $V_0 = 0$. Even if this is not the case, whenever V_0 is likely to be much smaller than $N_{\rm nom}$ then setting $V_0 = 0$ will hardly affect the estimation process.

C Parameter Estimation and Simulation Procedures

C.1 Estimation approaches

From the discussion in the previous, and prior to the onset of any drivers other than background surveillance and isolation/treatment rates, the set of parameters that we likely need to directly estimated from fitting our SCLAIV model to an initial COVID-19 incidence outbreak times series of say 30 or 40 points is

$$\underline{\text{Parameters estimated by fitting model output to incidence data:}} \quad \{\kappa, \pi_{\text{suc}}, C_0, I_0\} \tag{C.1}$$

Once various drivers are turned on (e.g. social distancing, quarantining), or go into switching mode (e.g. surveillance), then additional parameters will need to be fitted as discussed in the main text.

Beyond our initial fit, we assumed that one or more of the drivers came into operation, either implicitly (e.g., social distancing) or explicitly (increase in surveillance and quarantine rates) at points in time after the start of the outbreak. The parameters defining these emerging implicit drivers could be estimated by fitting model output to incidence data streams beyond those used to fit the basic SCLAIV process, while the parameters associated with the implemented explicit drivers will come from information on the application of these drivers to the population.

When estimating these drivers from incidence data beyond the initial data used to estimate the basic SCLAIV parameters, as a first tack driver x for example could be regarded being constant once switched on. In this case, in Eq. A.20 we set $\delta_{\text{x}\,\infty} = \delta_{\text{x}\,0}$, nominally set $\sigma_{\text{x}} = 1$ and $t_{\text{x}\,1/2} = 1000$ say, and then estimate the two parameters $t_{\text{x}\,0}$ and $\delta_{\text{x}\,0}$. If this does not provide a good enough fit, we can more general fit all five parameters $\left\{t_{\text{x}\,0}, \delta_{\text{x}\,0}, \delta_{\text{x}\,\infty}, t_{\text{x}\,1/2}, \sigma_{\text{x}}\right\}$ that define the form depicted in Eq. A.20, or all but one of the parameters if we set $\sigma_{\text{x}} = 2$ for a gradual switch, $\sigma_{\text{x}} = 5$ for an intermediate switch, or $\sigma_{\text{x}} = 20$ for an abrupt switc (Fig. A.2).

C.2 Forecasting and Scenario Analyses

One can usefully identify three time periods of interest associated with parameter estimation, validation (either informal or undertaking a statistically rigorous evaluation), and forecasting: i.e., $[0, t_{\text{est}}]$, $[t_{\text{est}}, t_{\text{data}}]$, and $[t_{\text{data}}, t_{\text{finish}}]$, respectively, where $t_{\text{est}} \leq t_{\text{data}}$ is the interval over which the parameter estimation procedure is conducted, t_{data} is the length of the data time series and t_{finish} is the end of the forecast interval. Although we expect t_{finish} to typically be greater than t_{data}, sometimes we may run a forecast on $[t_{\text{est}}, t_{\text{finish}}]$, where t_{finish} may be less than or equal to t_{data}. More generally, the onset of the forecasting interval may be different from t_{est} or t_{data}, which is why we have provide in NMB-DASA for a $t_{\text{forecast\_onset}}$ value to be selected on the forecasting page. Thus, it will always be the case that forecasts are made on the interval $[t_{\text{forecast\_onset}}, t_{\text{finish}}]$, where $t_{\text{forecast\_onset}}$ can be set to any value we like that is less than t_{finish}. Forecasting studies may be undertaken by generating ensembles of stochastic runs using a combination of fixed and MLE parameters. It may also be undertaken by substituting MCMC generated parameters, estimated over an interval that differs from the original estimation interval, for one or more of the MLE parameters, as discussed next.

C.3 MCMC method and results

We performed a Markov chain Monte Carlo (MCMC) procedure to fit the model parameters $\theta = \{$`SocDist_const, SocRel_const`$\}$ to the Israel incidence data D, considering the data points from day 128 to day 166. To perform this procedure, we used the R package `MCMCpack` and in particular the function `MCMCmetrop1R` (Metropolis Sampling from User-Written R function). We assumed a log-normal distribution as a prior distribution, while the log-likelihood function was calculated using a Poisson probability distribution [7]:

$$\ln \mathcal{L}(D|\theta) = \sum_{i=t_i}^{t_f} \ln \left(\frac{\lambda_i^{x_i} e^{-\lambda_i}}{x_i!} \right) = \sum_{i=t_i}^{t_f} \left(x_i \ln \lambda_i - \lambda_i - \ln(x_i!) \right) \tag{C.2}$$

where x_i are the data points, λ_i the generated values, t_i and t_f equal to 128 and 166 respectively. To calculate $\ln(x_i!)$, we used Lanczos approximation of the gamma function, as described in [16].

R code

We now present the R code used to run the MCMC procedure. We start by loading the package `MCMCpack`:

```
library(MCMCpack)
```

We then define the function which returns the log-likelihood, calculated as shown in Eq. C.2, considering the data between day 128 and day 166. Since the 2 considered parameters have to be positive values, we use a log-transformation for the sampling and then the exponential function to calculate the new proposed values. We do not declare the data and the final time as input since those are stored in the Numerus Model Builder (NMB) model, which was built for our SCLAIV+D+response model following the methods discussed in [11]

Computing the log-likelihood requires interaction between R and the NMB platform, since the latter contains the simulation that produces the MLE as the value of its ErrorFit term. We use a feature of the NMB platform that implements an API for enabling communication with the platform from other applications such as R. This API uses a simple dynamic translator and execution engine on a language consisting of a sequence of strings. This language, *Blackbox Command Language* (BCL) has a set of 20 opcodes including `reset`, `run`, `run_to`, `continue`, `get xxx` and `set xxx yyy` that are sufficiently expressive for the steps required to perform the Numerus computation.

We require a BCL script that performs a simulation up to day 128 with the MLE parameters enabled, following which the latter are turned off and replaced by MCMC proposals, and the simulation continued to its proscribed stopping point at day 166. The definition below shows how such a script is constructed in R using the paste command. The commands `set SocDistancing 1` and `set SocRelaxation 1` duplicates the parameter set related to social distancing and social relaxation used to obtain the fit with the sample data. We run this to day 128 using `run_to 128`. This part of the run follows the same trajectory as the fit, thus reaching the appropriate initial system state to start forecasting at day 128. At this point we turn off the switches for social distancing and social relaxation (`set SocDistancing 0`, `set SocRelaxation 0`), replace their roles in the simulation with SocDist_const and SocRel_const values drawn from the MCMC

proposal (`set SocDist_const` *sd*, `set SocRel_const` *sr*), and run the simulation until day 166 (`continue`). Finally we return the value of ErrorFit as the value of the script (`get ErrorFit`). The function `numerus(script)` performs the necessary marshalling of script and result between R and Numerus and returns the required value.

```
loglikelihood <- function(theta) {
  sd = exp(theta[1])
  sr = exp(theta[2])
  script <- paste(sep=" ", "reset","set SocDistancing 1",
             "set SocRelaxation 1","run_to 128",
             "set SocDistancing 0","set SocRelaxation 0",
             "set SocDist_const", sd,
             "set SocRel_const", sr,
             "continue", "get ErrorFit")
  errfit = numerus(script)
  return(-errfit)
}
```

Note that we return -ErrorFit since the calculation in the simulator evaluates $-\ln \mathcal{L}(D|\theta)$.

Since the parameters are sampled in log-scale, also for the log-prior calculation we consider the exponential function.

```
logprior <- function(theta){
  sd = exp(theta[1])
  sr = exp(theta[2])
  sdprior = dlnorm(sd,meanlog = -2.683269, sdlog = 1.061137, log=TRUE)
  srprior = dlnorm(sr,meanlog = -5.870255, sdlog = 0.5593254, log=TRUE)
  return(sdprior+srprior)
}
```

We then set up a function called `go` to facilitate the set up for the MCMC procedure. Within the function `go`, we called the function `MCMCmetrop1R` from the R package `MCMCpack`. This function requires different input: the function `fun` which returns the calculation of the log-posterior (sum of log-likelihood and log-prior, as a function of the chosen parameters), while `tune` is the tuning vector for the sampling (standard deviation of the proposal distribution). `theta.init` sets up the initial parameters, which will be considered in log-scale for the sampling, but passed to the simulator using the exponential function. The `burnin` defines the burn-in period while `mcmc` sets the number of iterations after the burn-in period. `verbose` defines how often the progress of the sampler is printed in the console. We also set `Logfun` as TRUE, since we are working with the log-posterior, `V` as NULL and the thinning interval to 10. This function will return the MCMC output (log-posterior sample) for each parameter.

```
go <- function(burnin, mcmc, verbose, tune) {
  post_sample <- MCMCmetrop1R(
  fun = function(theta){return(loglikelihood(theta)+logprior(theta))},
        tune=tune, theta.init = log(startvalue), burnin = burnin,
        mcmc = mcmc, verbose=verbose, logfun = TRUE, V=NULL, thin = 10)
  return(post_sample)
}
```

We then declare all the necessary input for the function go.

```
startvalue <- c(0.12,0.0033)
burnin = 0
mcmc = 500000
verbose= 10000
tune=c(0.55,0.15)
```

Note that we set the burn-in period equal to 0, to be able to choose an appropriate value for this parameter after the procedure terminates. In our case, we selected 2000 as burn-in.

```
post_sample <- go(burnin=burnin, mcmc=mcmc, verbose=verbose, tune=tune)
```

If we plot the output of the go function, we get information about the log transformation of the parameters (since Logfun is set as TRUE). To consider the plots and the distribution of the actual parameters, we will use the exponential function.

```
post_sample <- exp(post_sample)
```

MCMC results

In Figure C.1 we show the resulting trajectories of the MCMC procedure and the distribution of the resulting values. The acceptance rate was equal to 45.2%. To conclude, we show the cross-correlation between the two parameters in Figure C.2.

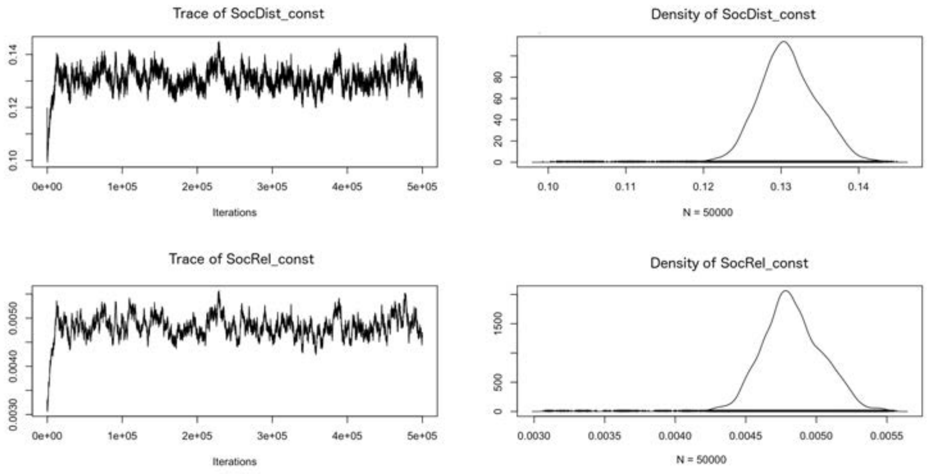

Figure C.1: MCMC output and distribution of the parameters SocDist_const (top) and SocRel_const (bottom).

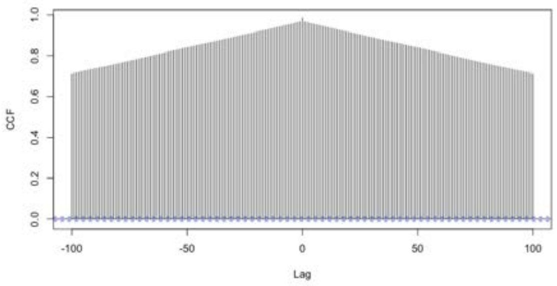

Figure C.2: Cross-correlation between the parameters SocDist_const and SocRel_const (calculated using the function `ccf` of R package `stats`). Value for lag 0 equal to 0.984.

D Additional Figures

D.1 Israel

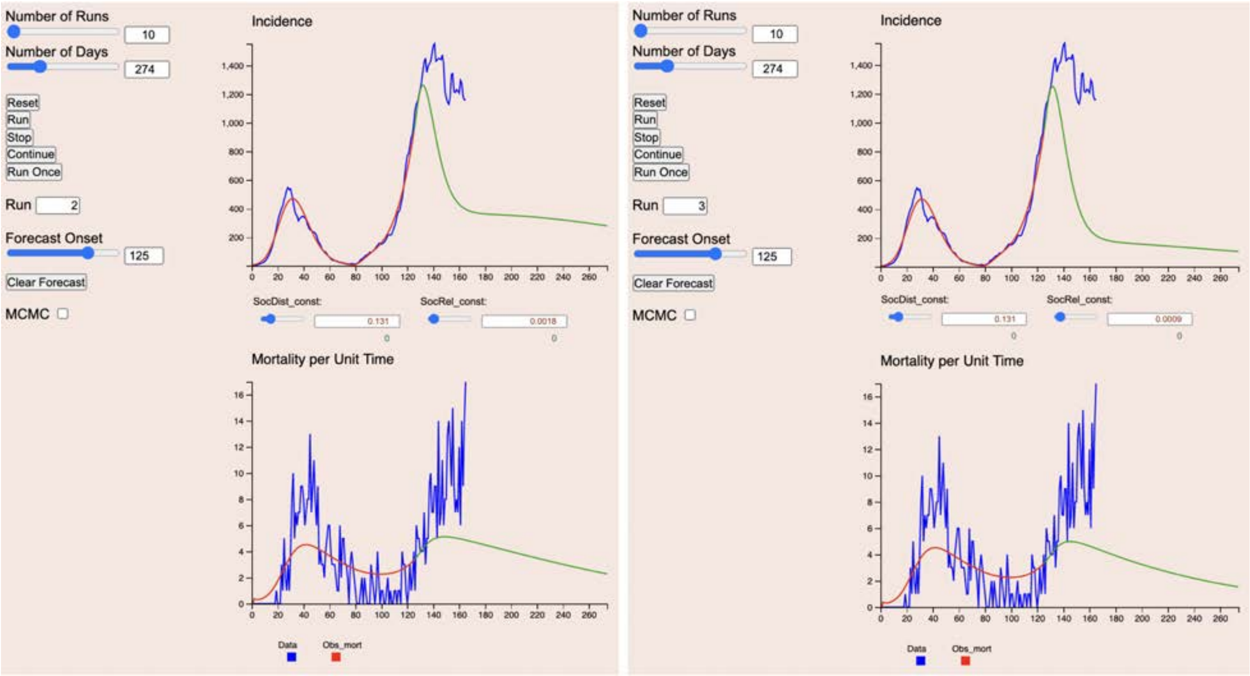

Figure D.1: Deterministic forecasts of Israeli epidemic on [125,274]. Cf. Fig. 9 in main text. Note that the values for `Soc_rel` of 0.0018 and 0.0009 represent 10% (left panel) and 5% (right panel) respectively of the value of 0.018 for `Soc_rel`(125), as discussed in the main text.

D.2 South Africa

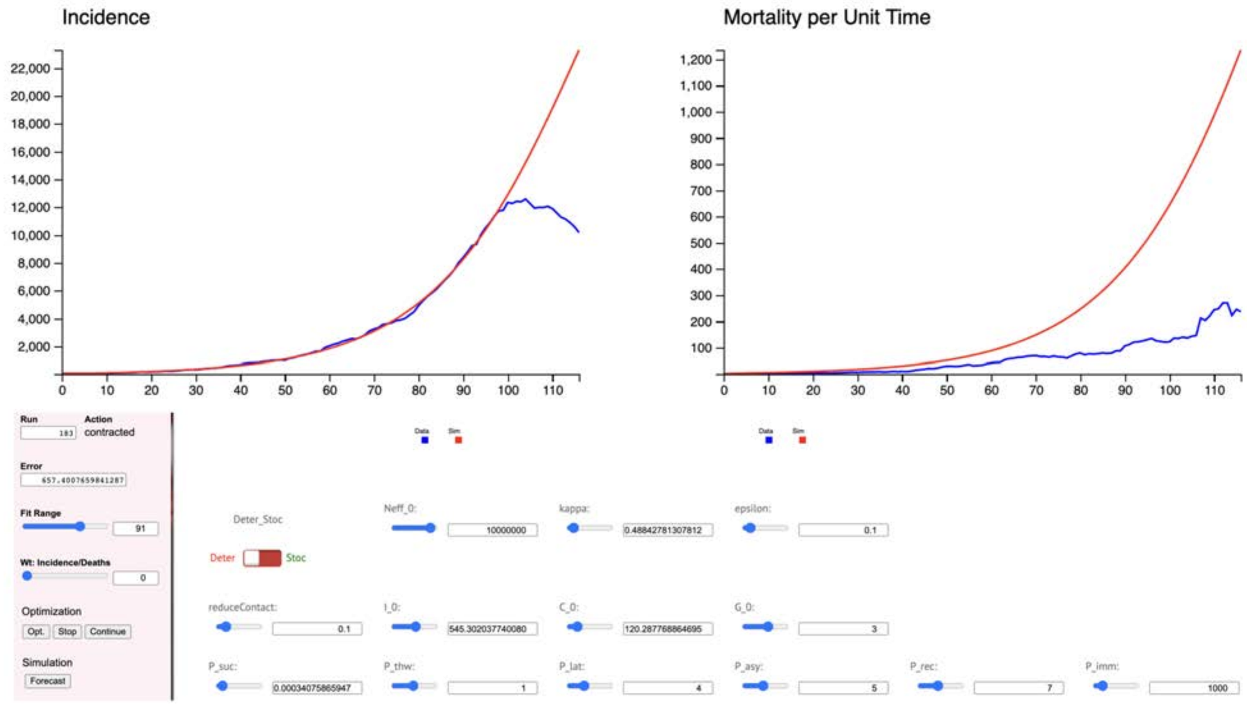

Figure D.2: MLE values and mortality simulation for main text Fig. 10C.

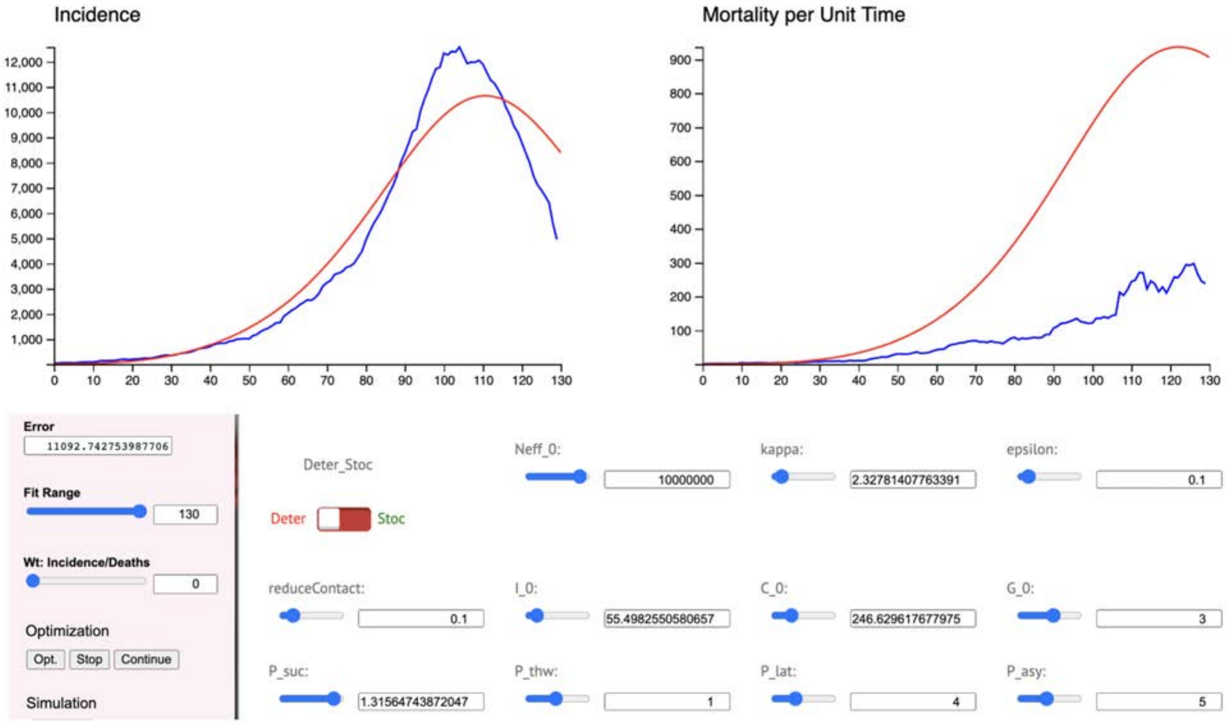

Figure D.3: MLE values and mortality simulation for main text Fig. 10D.

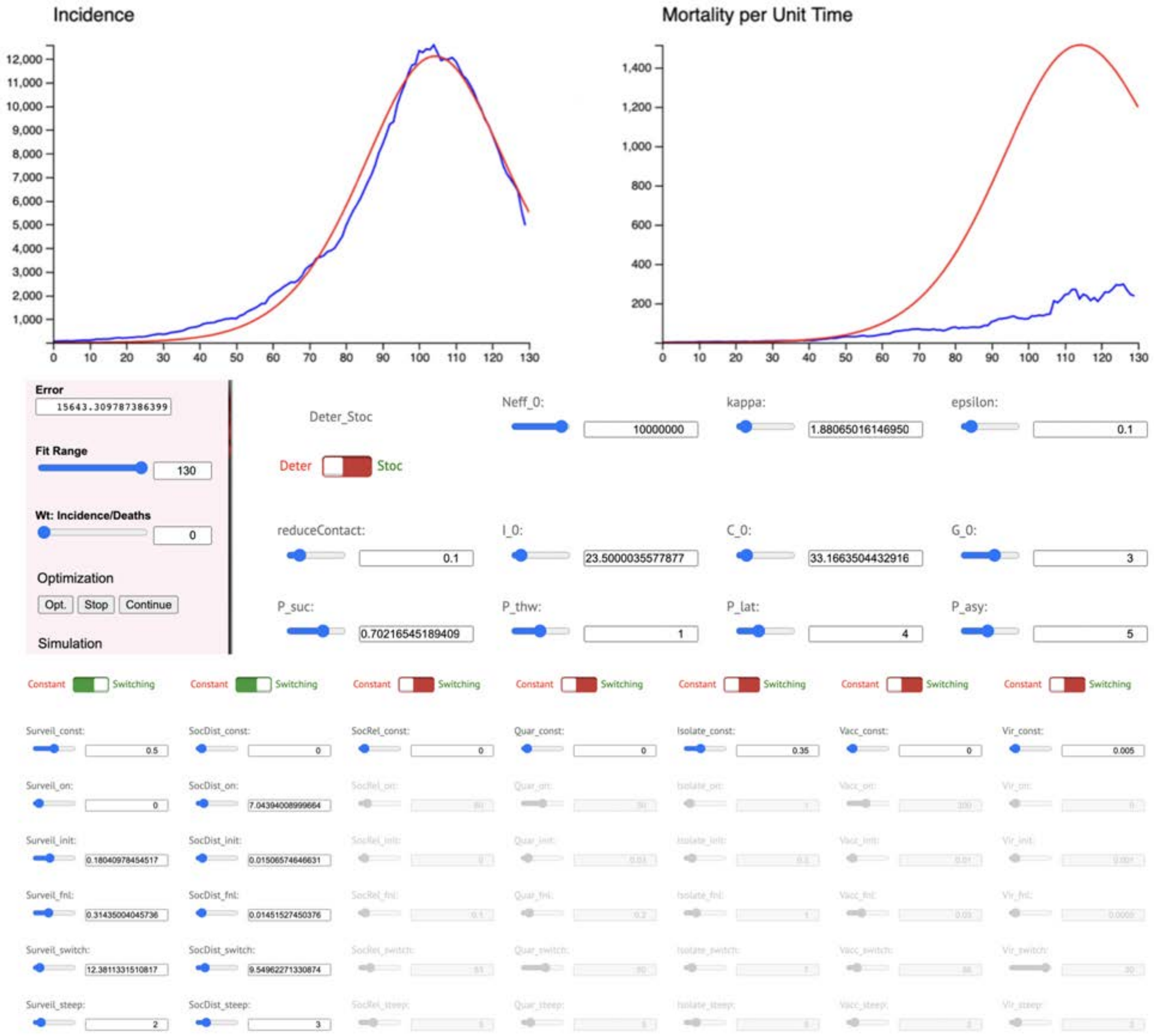

Figure D.4: MLE values and mortality simulation for main text Fig. 10E.

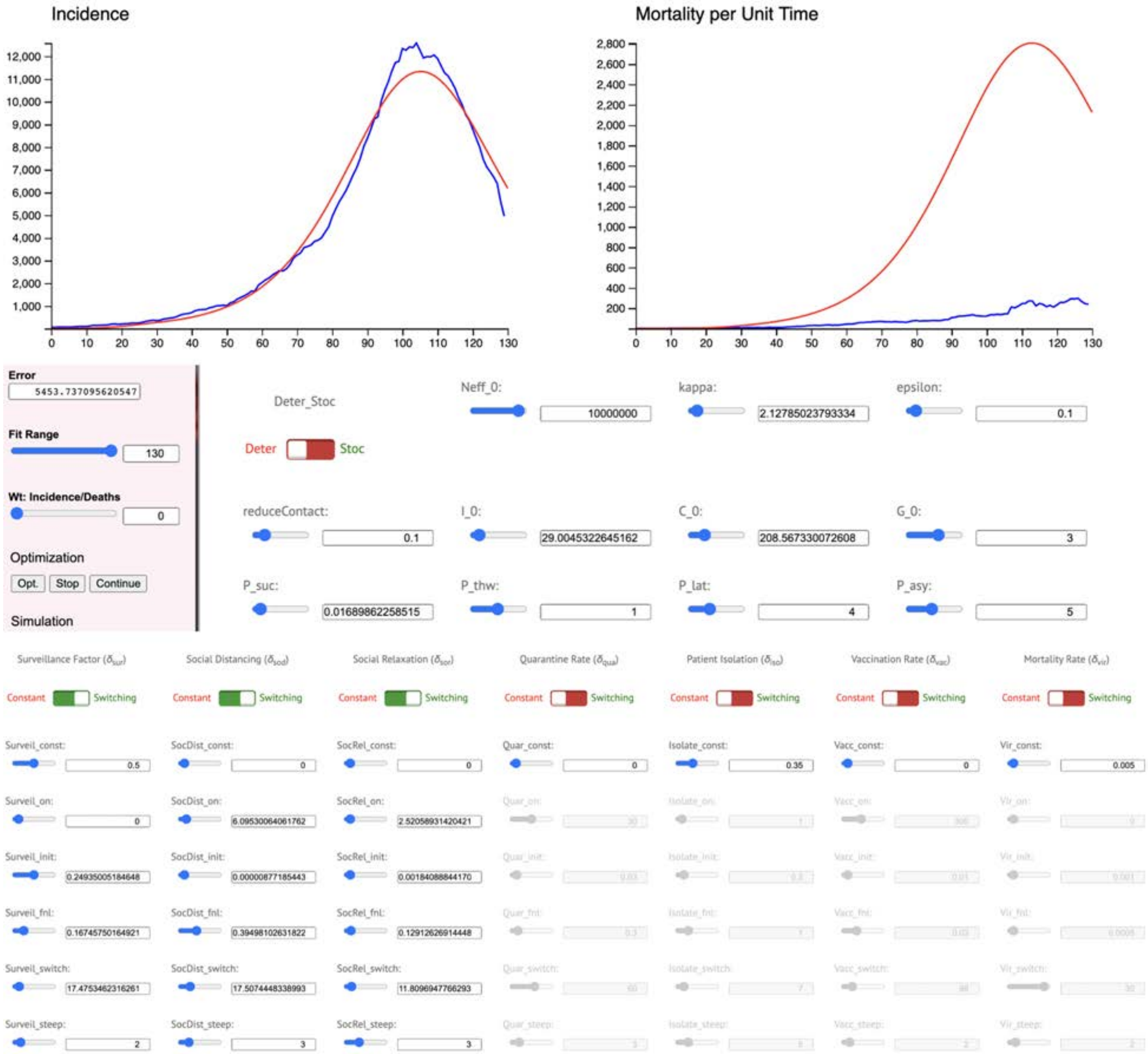

Figure D.5: MLE values and mortality simulation for main text Fig. 10F.

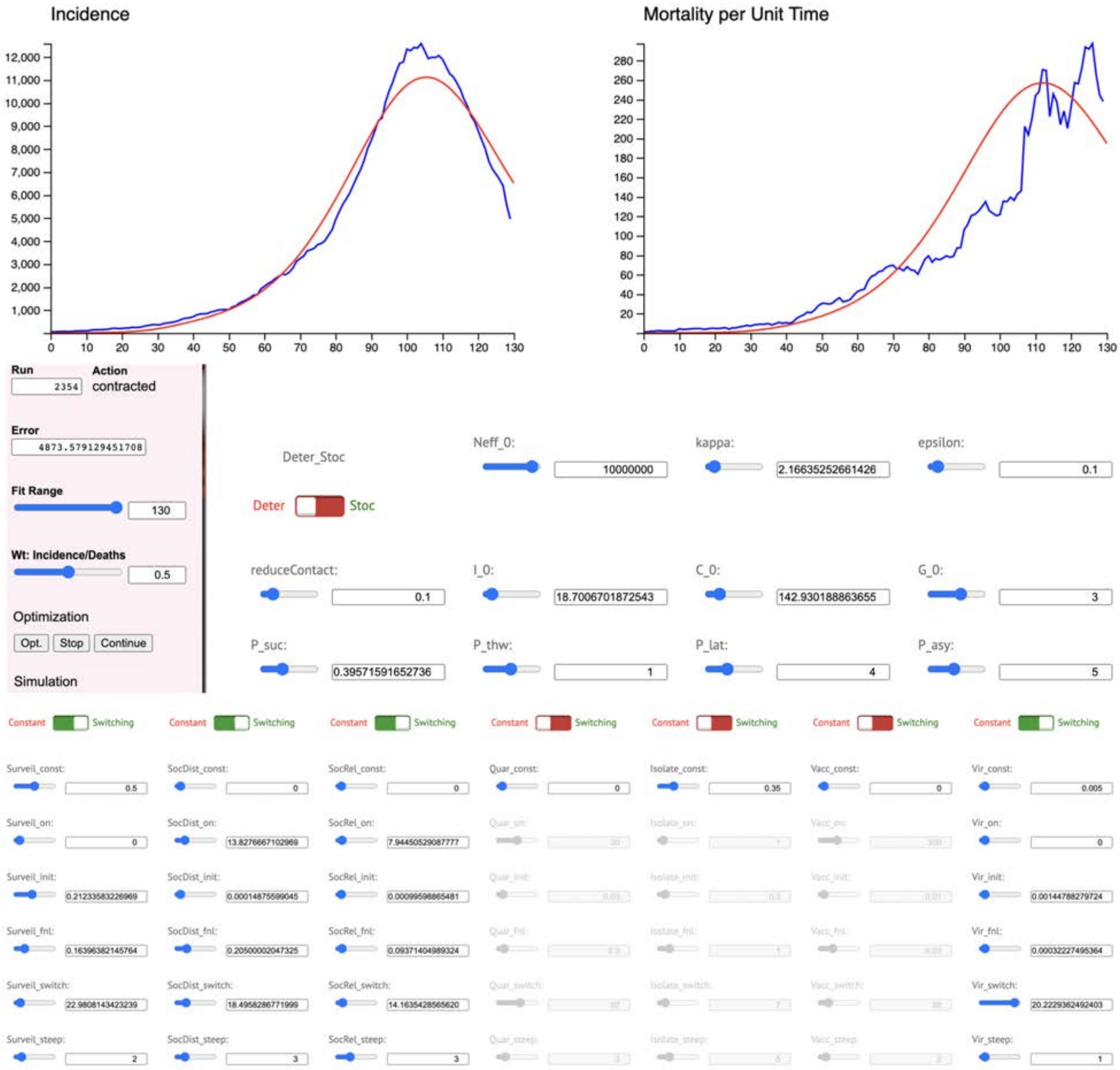

Figure D.6: MLE values for main text Figs. 10G1 and G2.

D.3 England

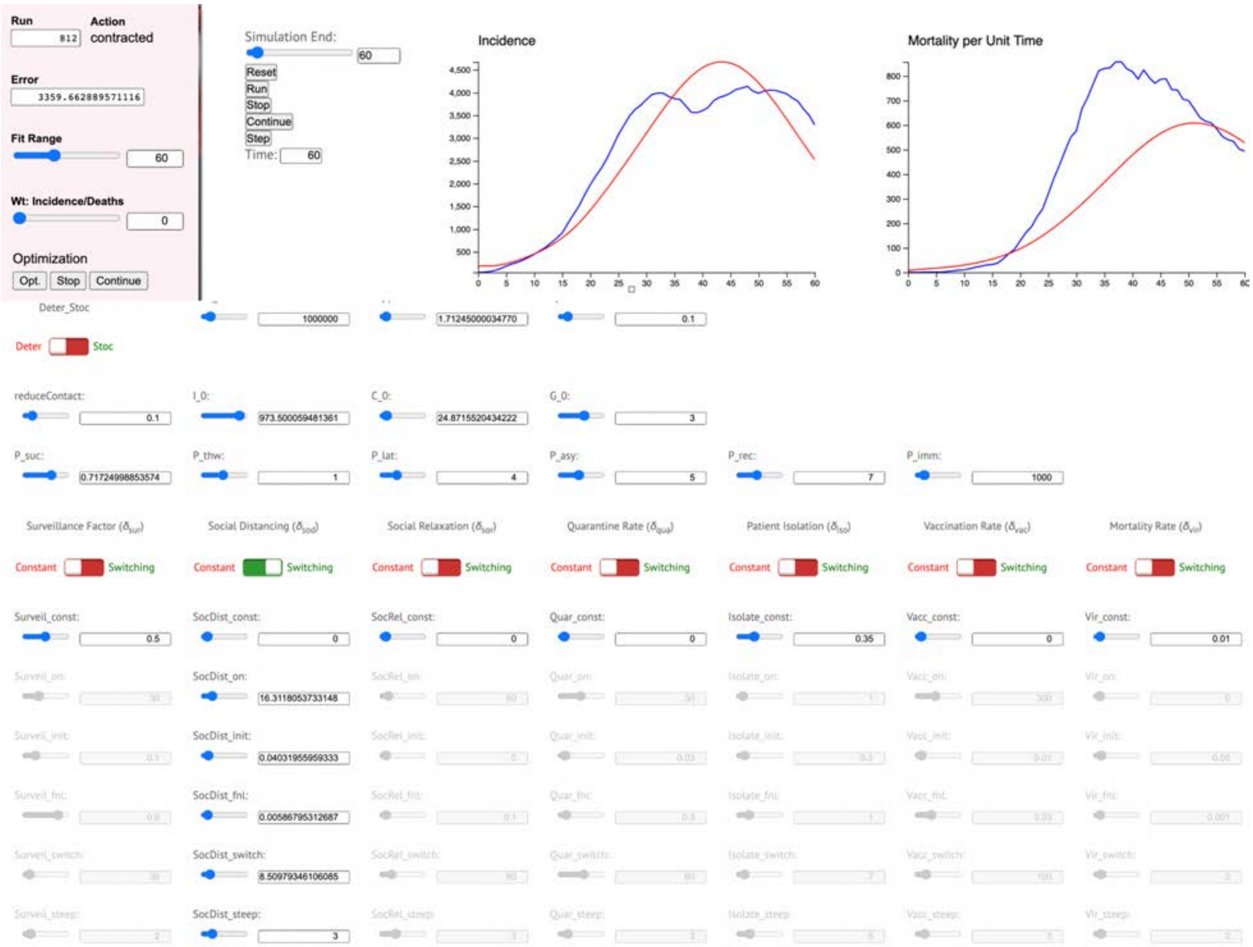

Figure D.7: MLE values and mortality simulation for main text Fig. 11C.

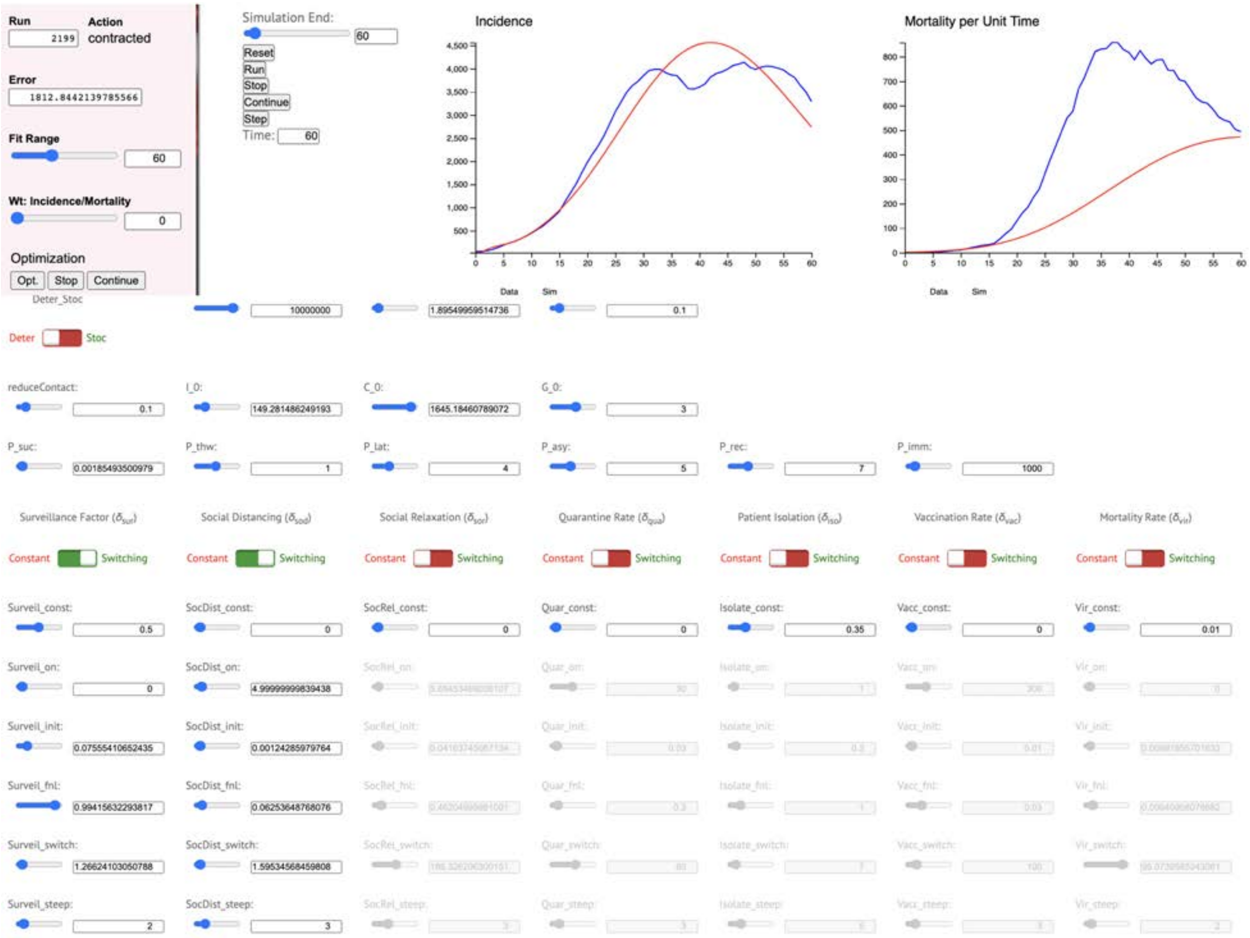

Figure D.8: MLE values and mortality simulation for main text Fig. 11D.

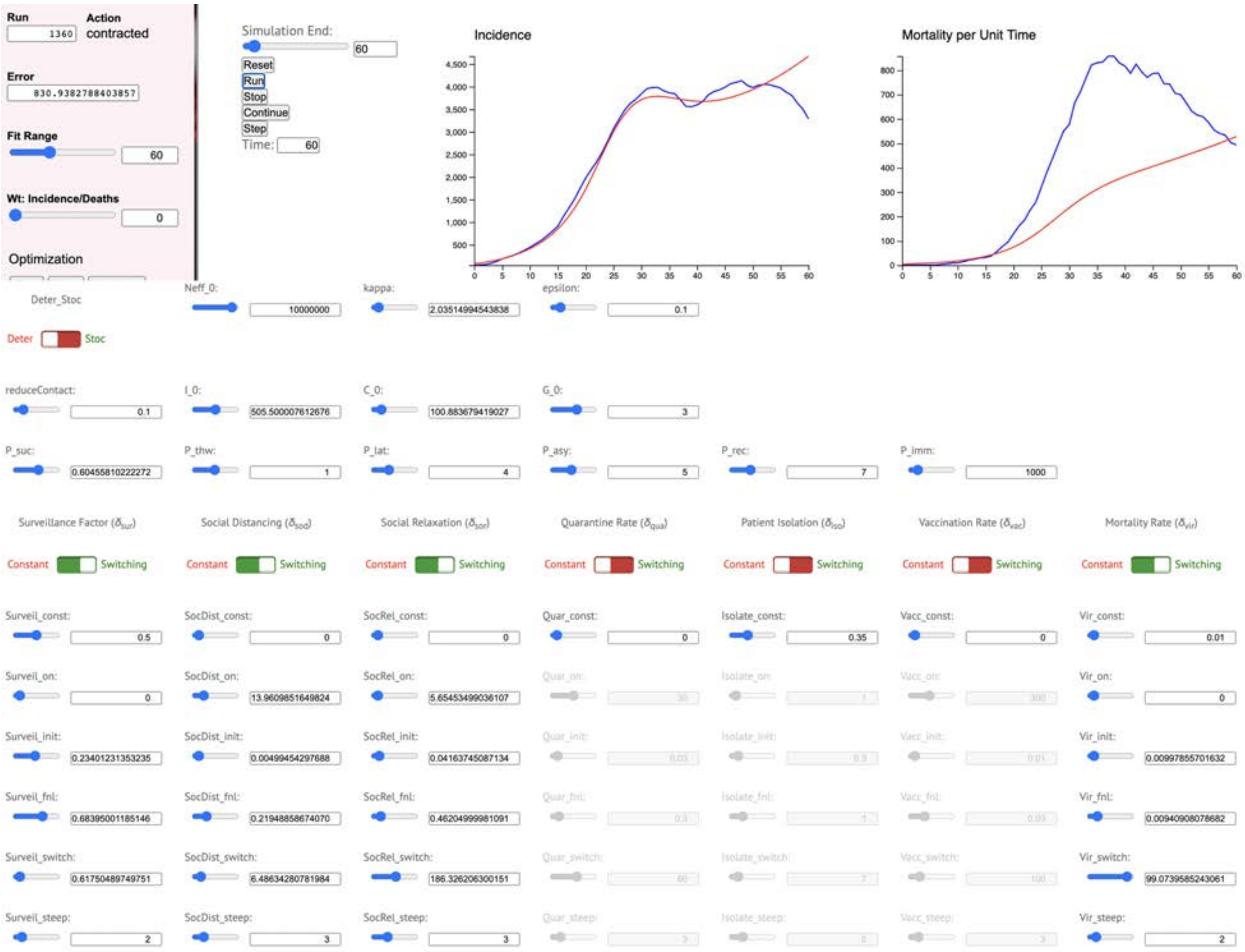

Figure D.9: MLE values and mortality simulation for main text Fig. 11E.

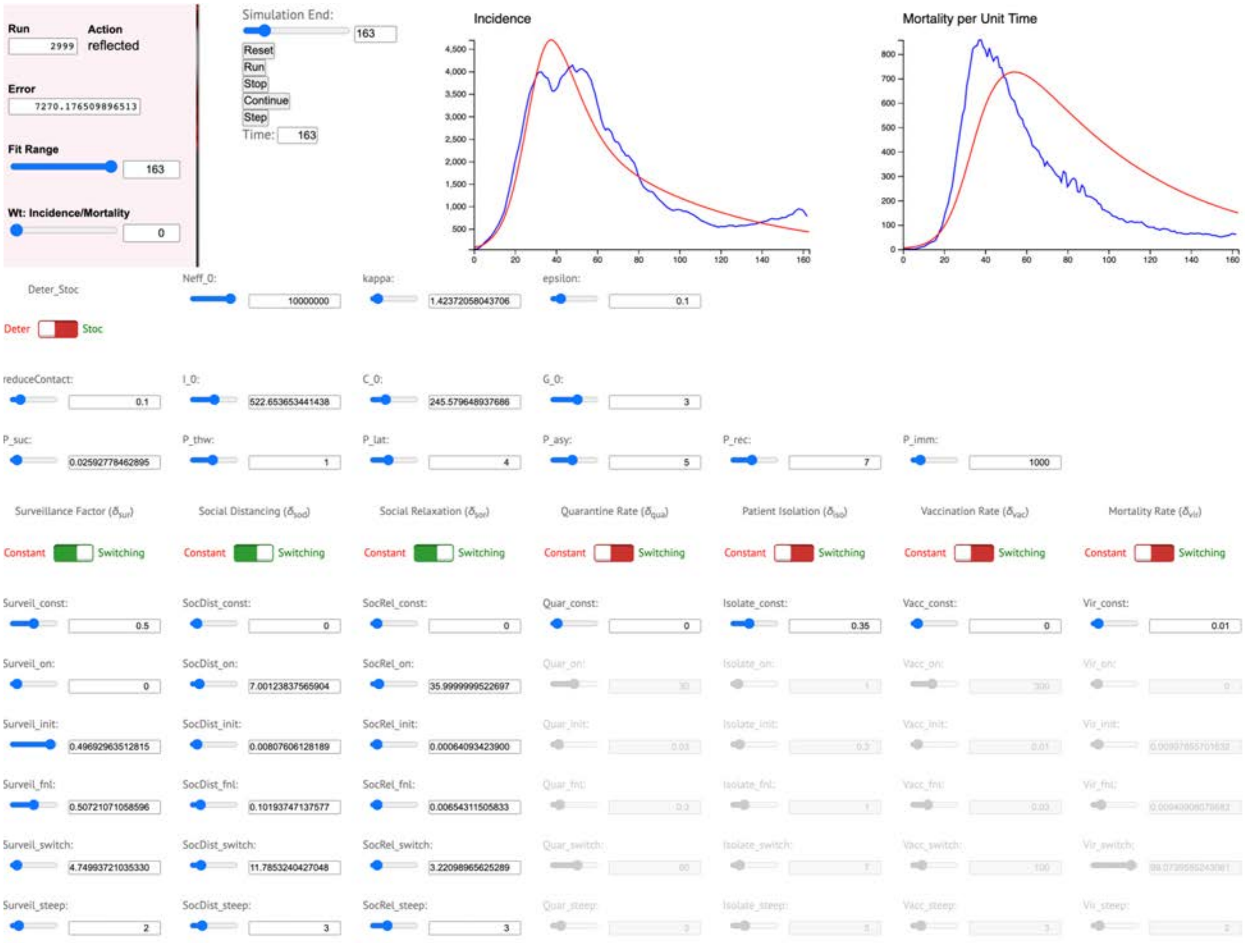

Figure D.10: MLE values and mortality simulation for main text Fig. 11F.

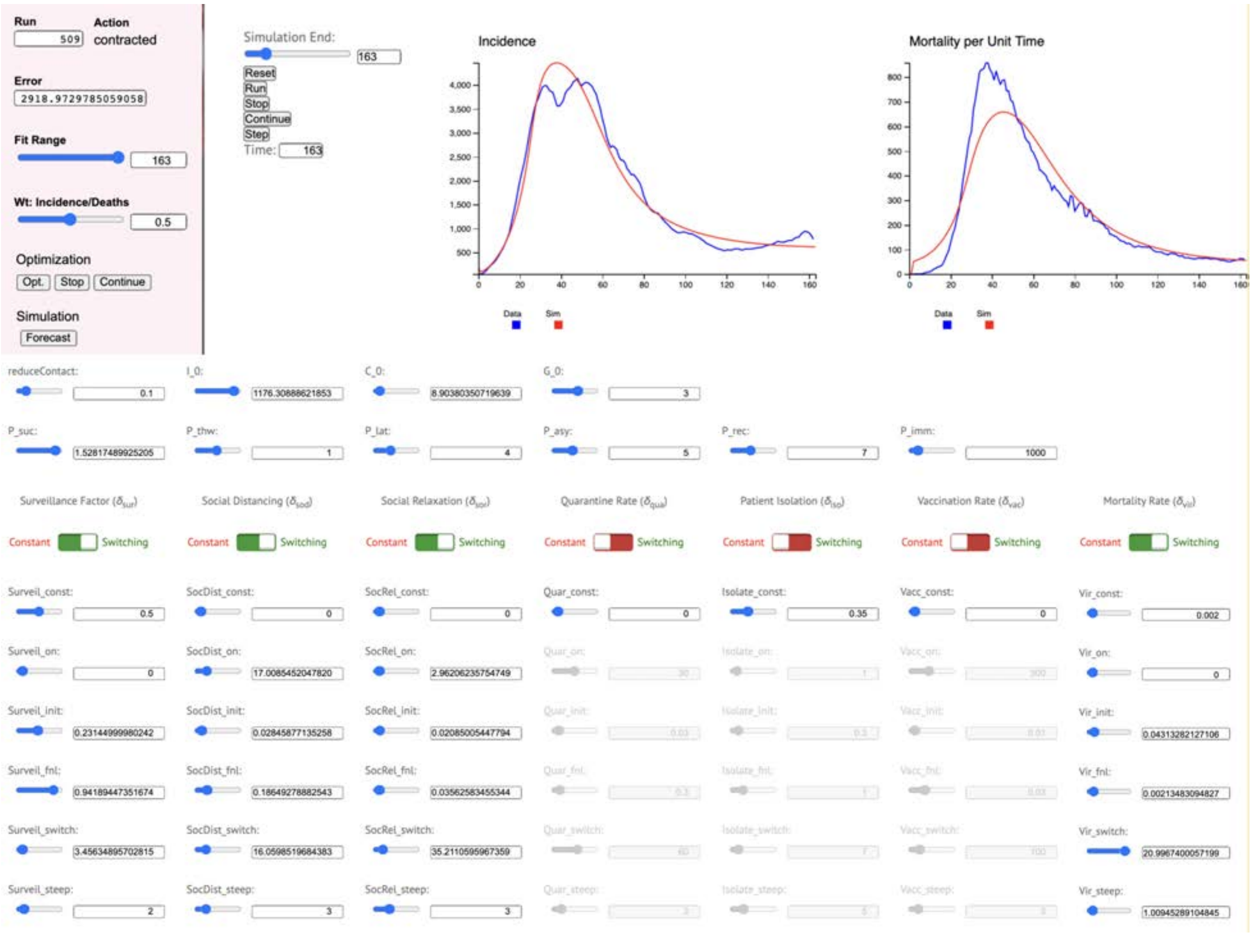

Figure D.11: MLE values for main text Figs. 11G1 and G2.

References

[1] Kathleen A Alexander, Colin J Carlson, Bryan L Lewis, Wayne M Getz, Madhav V Marathe, Stephen G Eubank, Claire E Sanderson, and Jason K Blackburn. The ecology of pathogen spillover and disease emergence at the human-wildlife-environment interface. In *The connections between ecology and infectious disease*, pages 267–298. Springer, 2018.

[2] Christian L Althaus. Estimating the reproduction number of ebola virus (ebov) during the 2014 outbreak in west africa. *PLoS currents*, 6, 2014.

[3] Michael H Cortez and Joshua S Weitz. Distinguishing between indirect and direct modes of transmission using epidemiological time series. *The American Naturalist*, 181(2):E43–E52, 2013.

[4] Jonas Dehning, Johannes Zierenberg, F Paul Spitzner, Michael Wibral, Joao Pinheiro Neto, Michael Wilczek, and Viola Priesemann. Inferring change points in the spread of covid-19 reveals the effectiveness of interventions. *Science*, 2020.

[5] Neil Ferguson, Daniel Laydon, Gemma Nedjati Gilani, Natsuko Imai, Kylie Ainslie, Marc Baguelin, Sangeeta Bhatia, Adhiratha Boonyasiri, ZULMA Cucunuba Perez, Gina Cuomo-Dannenburg, et al. Report 9: Impact of non-pharmaceutical interventions (npis) to reduce covid19 mortality and healthcare demand. *Imperial College of Science, Technology and Medicine*, 2020.

[6] Wayne M Getz. A hypothesis regarding the abruptness of density dependence and the growth rate of populations. *Ecology*, 77(7):2014–2026, 1996.

[7] Wayne M Getz and Eric R Dougherty. Discrete stochastic analogs of erlang epidemic models. *Journal of biological dynamics*, 12(1):16–38, 2018.

[8] Wayne M Getz, Jean-Paul Gonzalez, Richard Salter, James Bangura, Colin Carlson, Moinya Coomber, Eric Dougherty, David Kargbo, Nathan D Wolfe, and Nadia Wauquier. Tactics and strategies for managing ebola outbreaks and the salience of immunization. *Computational and mathematical methods in medicine*, 2015, 2015.

[9] Wayne M Getz and James O Lloyd-Smith. Basic methods for modeling the invasion and spread of contagious diseases. *DIMACS Series in Discrete Mathematics and Theoretical Computer Science*, 71:87, 2006.

[10] Wayne M Getz and John Pickering. Epidemic models: thresholds and population regulation. *The American Naturalist*, 121(6):892–898, 1983.

[11] Wayne M Getz, Richard Salter, Oliver Muellerklein, Hyun S Yoon, and Krti Tallam. Modeling epidemics: A primer and numerus model builder implementation. *Epidemics*, 25:9–19, 2018.

[12] Hongbin Guo, Michael Y Li, and Zhisheng Shuai. Global stability of the endemic equilibrium of multigroup sir epidemic models. *Canadian applied mathematics quarterly*, 14(3):259–284, 2006.

[13] Ghassan Hamra, Richard MacLehose, and David Richardson. Markov chain monte carlo: an introduction for epidemiologists. *International journal of epidemiology*, 42(2):627–634, 2013.

[14] Herbert W Hethcote. The mathematics of infectious diseases. *SIAM Review*, 42(4):599–653, 2000.

[15] Hamish McCallum, Nigel Barlow, and Jim Hone. How should pathogen transmission be modelled? *Trends in ecology & evolution*, 16(6):295–300, 2001.

[16] William H. Press, Saul A. Teukolsky, William T. Vetterling, and Brian P. Flannery. *Numerical Recipes in C (2nd Ed.): The Art of Scientific Computing*. Cambridge University Press, USA, 1992.

[17] Tingzhe Sun and Dan Weng. Estimating the effects of asymptomatic and imported patients on covid-19 epidemic using mathematical modeling. *Journal of medical virology*, 2020.

[18] Neeltje Van Doremalen, Trenton Bushmaker, Dylan H Morris, Myndi G Holbrook, Amandine Gamble, Brandi N Williamson, Azaibi Tamin, Jennifer L Harcourt, Natalie J Thornburg, Susan I Gerber, et al. Aerosol and surface stability of sars-cov-2 as compared with sars-cov-1. *New England Journal of Medicine*, 382(16):1564–1567, 2020.

[19] Erik M Volz, Katia Koelle, and Trevor Bedford. Viral phylodynamics. *PLoS Comput Biol*, 9(3):e1002947, 2013.

[20] Wendi Wang. Backward bifurcation of an epidemic model with treatment. *Mathematical biosciences*, 201(1-2):58–71, 2006.

[21] Lander Willem, Frederik Verelst, Joke Bilcke, Niel Hens, and Philippe Beutels. Lessons from a decade of individual-based models for infectious disease transmission: a systematic review (2006-2015). *BMC infectious diseases*, 17(1):612, 2017.
